# Judging the morality of utilitarian actions: How poor utilitarian accessibility makes judges irrational

**DOI:** 10.3758/s13423-016-1029-2

**Published:** 2016-04-27

**Authors:** Petko Kusev, Paul van Schaik, Shrooq Alzahrani, Samantha Lonigro, Harry Purser

**Affiliations:** 1 Department of Psychology, Kingston University London, London, KT1 2EE UK; 2 Department of Psychology, Teesside University, Middlesbrough, UK; 3 Department of Psychology, City University London, London, UK; 4 Department of Psychology, Sapienza University of Rome, Rome, Italy; 5 Department of Psychology, University of Nottingham, Nottingham, UK

**Keywords:** Utility, Moral dilemmas, Accessibility, Judgments, Rational choice

## Abstract

Is it acceptable and moral to sacrifice a few people’s lives to save many others? Research on moral dilemmas in psychology, experimental philosophy, and neuropsychology has shown that respondents judge utilitarian personal moral actions (footbridge dilemma) as less appropriate than equivalent utilitarian impersonal moral actions (trolley dilemma). Accordingly, theorists (e.g., Greene et al., 2001) have argued that judgments of appropriateness in personal moral dilemmas are more emotionally salient and cognitively demanding (taking more time to be rational) than impersonal moral dilemmas. Our novel findings show an effect of psychological *accessibility* (driven by partial contextual information; Kahneman, 2003) on utilitarian moral behavior and response time for rational choices. Enhanced accessibility of utilitarian outcomes through comprehensive information about moral actions and consequences boosted utility maximization in moral choices, with rational choices taking less time. Moreover, our result suggests that previous results indicating emotional interference, with rational choices taking more time to make, may have been artifacts of presenting partial information.

**Electronic supplementary material:**

The online version of this article (doi:10.3758/s13423-016-1029-2) contains supplementary material, which is available to authorized users.

Is it acceptable and moral to sacrifice a few people’s lives to save many others? *‘It is the greatest happiness of the greatest number that is the measure of right and wrong’.* With these words, the British philosopher Jeremy Bentham (1970) defined the nature of utilitarian actions: Behaviors judged as morally right only by virtue of their outcome (Bentham, 1970). From the utilitarian point of view, Bentham (1970) noted that is acceptable to sacrifice a small number of people’s lives to save a greater number because this results in greater utility (happiness) overall. In contrast, deontologists (e.g., Kant, 1959) have argued that it is not acceptable, because living is a fundamental right for everyone, and no one has the right to take that from anyone, regardless of any benefits that may arise from doing so. Research in psychology, experimental philosophy, and neuropsychology has revealed that moral judgments of the appropriateness of life-saving actions are not strictly utilitarian, but are influenced by the type of involvement (e.g., Greene, Sommerville, Nystrom, Darley, & Cohen, 2001; Greene & Haidt, 2002; Mikhail, 2007, 2009; Thomson, 1985). In particular, directly taking action (“personal action”) in scenarios (one person pushing another from the bridge in order to save several others, in the “footbridge dilemma”) was judged to be less appropriate than indirectly taking action (“impersonal action”) (a person “switching a mechanism,” killing one person in order to save several others, in the “trolley dilemma”).

Various theoretical attempts have been made to account for these behavioral differences in response to personal and impersonal dilemmas. Traditionally, moral- psychology theorists have focused on the role of emotional processes in moral judgments (Cushman, Young, & Hauser, 2006; Greene et al., 2001; Greene & Haidt, 2002; Haidt, 2001; Nakamura, 2013; Valdesolo & DeSteno, 2006). For instance, Greene and colleagues (2001; Greene & Haidt, 2002) found that respondents spent more time judging the appropriateness of personal moral actions than of impersonal actions. This result seems puzzling and surprising from a strict utilitarian perspective, given that the two dilemma types offer identical utility.

In an attempt to provide an account of the above result in terms of the relationship between implicit and explicit cognitive processes in moral judgments, Greene and colleagues (2001) proposed a dual-process theory of moral behavior, stating that moral judgments can be driven via both (i) implicit, fast, affective, and (ii) explicit, slow, controlled psychological mechanisms (Forbes & Grafman, 2010; Greene et al., 2001; Greene & Haidt, 2002; Greene, Nystrom, Engell, Darley, & Cohen, 2004; Moore, Clark, & Kane, 2008). In Greene’s view, the affective system is likely to be activated by “personal” moral considerations, while the cognitive system might favor utilitarian consequences and thus rational thinking. This proposal has been supported by behavioral experiments (Greene et al., 2001), testing utilitarian choices in a morally challenging situation, in which a trolley is riding a rail and – if it proceeds on its way – five people tied on the track will be killed. The participants were presented with two different opportunities: in the *trolley dilemm*a, to hit a switch and make the trolley change its track, killing one person tied to another rail, or to do nothing and let the five people die; similarly, in the *footbridge dilemma*, to push a person off the bridge and onto the tracks below where his large body will stop the trolley, saving the five people tied up onto the track, or to do nothing and let the five people die. The results show that people judge as *appropriate* sacrificing one person for the sake of five in the trolley dilemma, but judge as *inappropriate* sacrificing one person in order to save five in the footbridge dilemma.

According to the moral dual-process model, what makes the difference between the two types of dilemma is the degree of personal affective and cognitive involvement. Consistent with this model, participants took longer to accept (i.e., judge as appropriate) personal moral actions that would maximize utilitarian outcomes (rational moral judgments) than to reject such actions as inappropriate (irrational moral judgments) (Greene et al., 2001; Greene & Haidt, 2002). Greene and colleagues (2001) argued that when participants faced personal (footbridge-like) dilemmas in which one’s moral rules conflict with the outcomes, both affective and cognitive systems were recruited. The former would favor rejecting the actions for the sake of an internal moral principle; the latter would favor endorsing them, in the name of rationality. The conflict, then, between the two systems would result in increased response time when a participant faced a footbridge-like dilemma and made a rational judgment.

Crucially, almost all experimental studies based on Thomson’s (1985) paradigm have tended to use abstract moral dilemmas framed in such a way that the *accessibility* (Kahneman, 2003) of moral utilitarian actions and consequences is reduced, asking respondents to apparently put themselves into those cognitively challenging situations.

For example:

“…The only way to save the lives of the five workmen is to hit a switch near the tracks that will cause the trolley to proceed to the right, where the lone workman’s large body will stop the trolley. The lone workman will die if you do this, but the five workmen will be saved.

Is it appropriate for you to hit the switch in order to avoid the deaths of the five workmen?

Yes/No”

There are two striking issues in these commonly used descriptions of abstract moral dilemmas. First, although there is an explicit contextual account about the moral action and utilitarian consequences of saving the five workmen at the expense of the stranger, there is no corresponding account of saving the life of the stranger at the expense of the workmen. Hence, only 50 % of the moral scenario is contextually available – a *framing effect* (Kahneman, 2003; Tversky & Kahneman, 1981), where different representations of outcomes make some features of the situation more accessible and others less accessible, leading to systematically different decisions. Second, the appropriateness question itself further adds to this framing effect by requiring an assessment of appropriateness on only one of the two possible moral actions (“Is it appropriate for you to hit the switch in order to avoid the deaths of the five workmen?”). Given the well-established role of contextual framing effects in decision-making (FeldmanHall, Mobbs, Evans, Hiscox, Navrady, & Dalgleish, 2012; Tversky & Kahneman, 1981), findings and interpretation of utilitarian moral decision-making based on these commonly used scenarios are to be treated with caution.

For the current study, in an attempt to increase the accessibility of moral utilitarian actions and consequences – utilitarian accessibility – we have developed and de-biased abstract moral scenarios and questions used by researchers in psychology, experimental philosophy, and neuroscience.

For example:

“….The only way to save the lives of the five workmen is to hit a switch near the tracks that will cause the trolley to proceed to the right, where the lone workman’s large body will stop the trolley. The lone workman will die if you do this, but the five workmen will be saved. The only way to save the life of the lone workman is not to hit the switch near the tracks. The five workmen will die if you do this, but the lone workman will be saved.

Choose the option which is more appropriate for you:

Sacrifice one workman in order to save five workmen

or

Sacrifice five workmen in order to save one workman.”

First, we offer a new experimental approach to study moral dilemmas by eliminating confounding variables (see, e.g., McGuire et al., 2009), allowing the footbridge dilemma to be impersonal (switching mechanism) and for the trolley dilemma to be personal (to push the worker on the track). Second, to account for utilitarian accessibility we offer presentations of moral dilemmas by using both partial textual descriptions (commonly employed in utilitarian moral research) and novel full textual descriptions of moral actions and their consequences. Third, we further reduce differences in utilitarian accessibility by offering a choice question of appropriateness, which accounts for both utilitarian alternatives (and their consequences) in moral actions (rational and irrational choice). Accordingly, the results of the current study were expected to reveal an enhanced behavioral rationality for moral dilemmas with accessible utilitarian content, where a full textual description was provided about the initial state, action, and the consequences of the action.

## Experiment

### Method

#### Participants

According to power analysis with a significance level = .05, desired power = .80, and medium effect size (*f* ^*2*^ = .25), a total sample size of 136 was required. Participants were recruited through a recruitment service of online survey panels. A window of 7 days was set for data collection; after a week had passed, 299 people (170 females, 129 males) had taken part, meeting the required sample size. Mean age was 49 years (*SD* = 14.07). They took part individually and received a payment of £1. All participants were treated in accordance with the ethical standards of the British Psychological Society.

#### Materials and design

Each participant was given one of eight vignettes to read, involving a moral-dilemma scenario where the type of dilemma, action involvement, task instructions and questions were manipulated. The experiment accounted for utilitarian accessibility by presenting descriptive information about the moral dilemmas: (1) by partial text description and question only or (2) by full textual description and question, revealing all of the possible behavioral actions and consequences of the actions (see the supplementary materials).

An independent measures 2 × 2 × 2 design was employed, with independent variables *type of dilemma* (trolley dilemma or footbridge dilemma), *action involvement* (moral personal or moral impersonal), and *utilitarian accessibility* (partial text description and question or full text description [displayed information about the initial state, action, and consequences of the action] and question). The dependent variables were the choice of appropriateness of action (making a rational or irrational choice), study time (reading the scenarios), and response time. Based on the consequentialist theory of moral utilitarian judgment, in this experiment we defined a rational choice as one that saves the lives of five workmen rather than of another single workman, thereby maximizing the utility of the moral action that is taken and minimizing the disutility. The order of the response options (rational and irrational) was counterbalanced across participants.

#### Procedure

Instructions, scenario, and question were presented in an online computer-based experiment. Participants were presented with and required to read the instructions and one moral-dilemma scenario. Then (after clicking the “next” button), while the moral dilemma was still visible, the respondents were presented with a binary choice (between actions with rational or irrational utilitarian consequences) and required to choose the appropriate option for them.

### Results

**Fig. 1:**
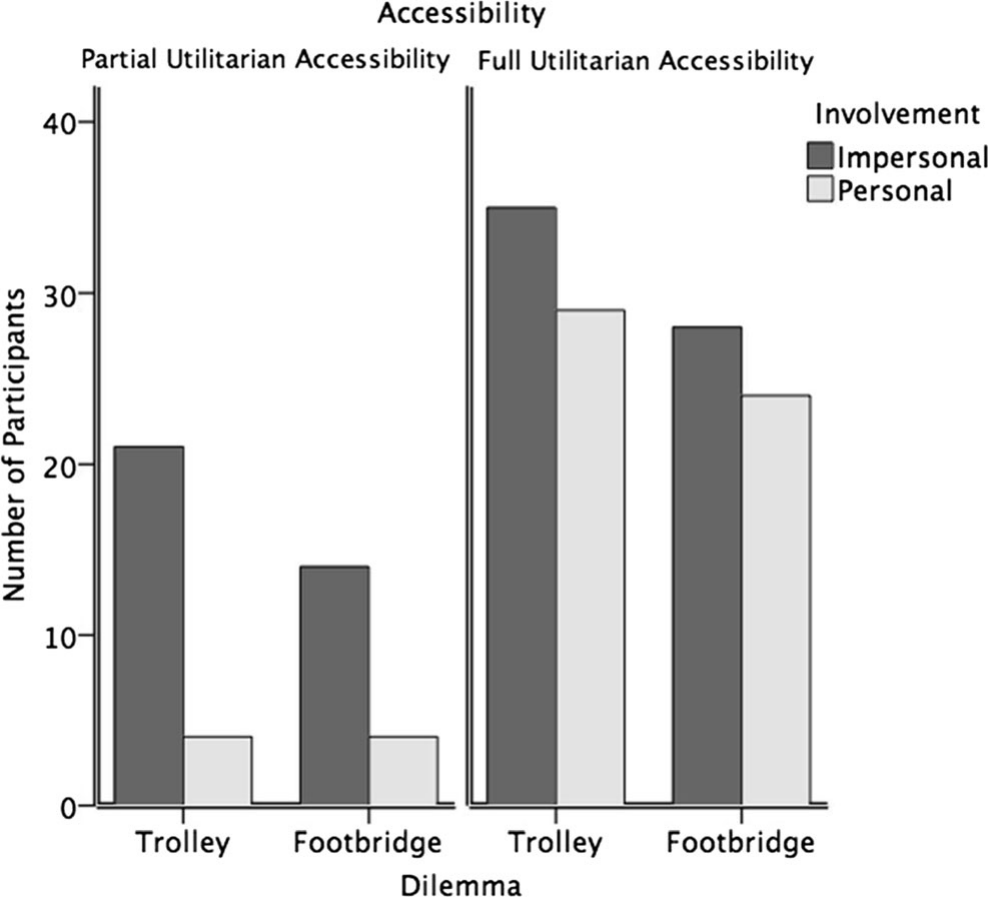
Frequencies of rational choices as a function of accessibility, involvement, and dilemma type

The effect of the independent variables on *choice* 1 was analyzed. Rational choices (choosing the option resulting in one death rather than five) were more commonly made when full information was presented and when an impersonal dilemma presented (Table 1 and Fig. 1): A logistic-regression model comprising all the main effects and interaction effects explained 38 % of variance, R_CS_^2^ = .38. The main effects of accessibility (partial information vs. full information), OR (odds ratio) = 31.67, 95 % confidence interval (CI) 3.95–254.08, and involvement (impersonal vs. personal), = 0.09, 95 % CI 0.03–0.31, were significant. However, neither the main effect of dilemma type, OR = 0.55, 95 % CI 0.22–1.37, nor any of the interaction effects, OR = 1.97, 95 % CI 0.35–10.97, for dilemma by involvement, OR = 0.24, 95 % CI 0.02–2.56, for dilemma by accessibility, OR = 1.79, 95 % CI 0.15–21.96, for involvement by accessibility, and OR = 1.43, 95 % CI 0.07–29.25, for involvement by accessibility were significant. Therefore, next a model with only the significant main effects of accessibility and involvement was analyzed. This explained 36 % of variance, R_CS_^2^ = .36. The main effects of accessibility, OR = 19.26, 95 % CI 10.00–31.11, and involvement, OR = 0.20, 95 % CI 0.10–0.37, remained significant. The odds of a rational choice were 19.26 times larger when a dilemma was presented with full information than when it was presented with reduced information. Furthermore, the odds of a rational choice were 0.20 times smaller when a dilemma involved a choice of a personal act (pushing the person) than when it involved an impersonal act (operating a switch without direct contact with the person).

**Table 1:** Choice as a function of involvement, accessibility, and dilemma type

Involvement	Accessibility (information)	Trolley		Footbridge	
		Irrational	Rational	Irrational	Rational
Impersonal	Partial	6 %	7 %	8 %	5 %
		(19)	(21)	(23)	(14)
	Full	0 %	12 %	2 %	9 %
		(1)	(35)	(6)	(28)
Personal	Partial	13 %	1 %	12 %	1 %
		(39)	(4)	(36)	(4)
	Full	2 %	10 %	4 %	8 %
		(5)	(29)	(11)	(24)

Figures are percentages with frequencies in brackets

*Study time* for a dilemma with full information was longer than when partial information was displayed; furthermore, when involvement was impersonal, time was longer than when it was personal (Table 2). A 2 × 2 × 2 analysis of variance (ANOVA) showed that the main effects of accessibility (partial vs. full information), *F*(1, 291) = 13.31, *p* < .001, ε^2^ = .04, and involvement (impersonal vs. personal), *F*(1, 291) = 5.33, *p* < .05, ε^2^ = .01, were significant, but neither the main effect of dilemma type nor any of the interaction effects, all *F* < 1, all ε^2^ = .01, were significant.

**Table 2:** Descriptives for study time by involvement, accessibility, and dilemma type

Involvement	Accessibility (information)	Tr	Trolley		Footbridge	
			*M*	*SD*	*M*	*SD*
Impersonal	Partial	T	3.23	0.50	3.29	0.60
		U	28.43	14.27	31.38	17.28
	Full	T	3.40	0.53	3.43	0.54
		U	34.55	20.65	36.46	27.16
Personal	Partial	T	3.12	0.44	3.15	0.46
		U	21.63	8.74	25.56	9.85
	Full	T	3.36	0.47	3.35	0.50
		U	30.01	12.49	32.10	16.86

The frequency distribution of study time was positively skewed and this was considerably improved by logarithmic transformation

*Tr* transformation, *T* logarithmically transformed, *U* untransformed (original)

**Fig. 2:**
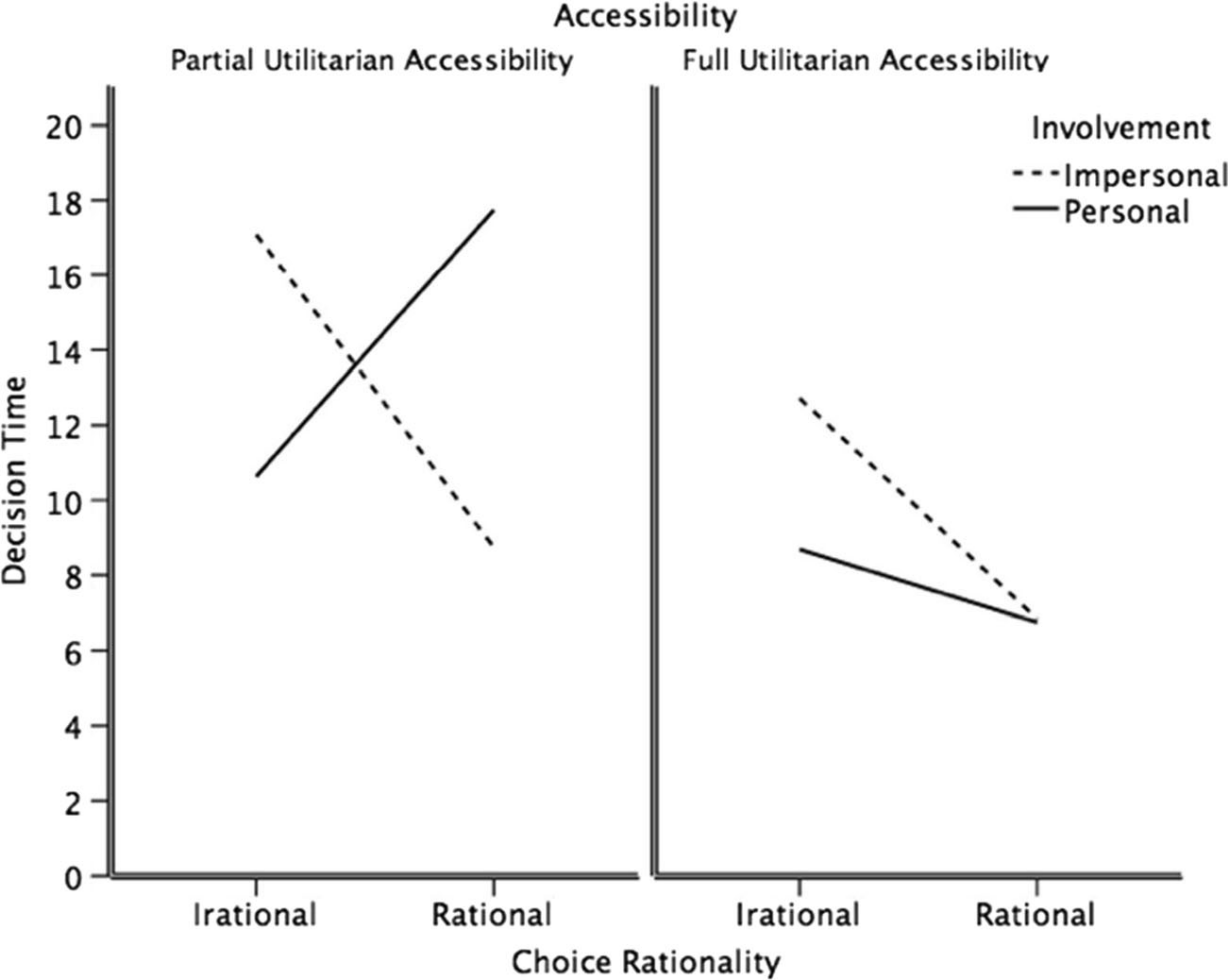
Mean response time as a function of accessibility, involvement, and choice rationality (time in seconds)

In contrast, *response time* for a dilemma with full information was shorter than when partial information was displayed (Table 3), *t* (297) = 5.57, *r* = .31, *p* < .001. Further analysis examined Greene and colleagues’ (2001) claim that “emotional interference” produces a longer response time for emotionally incongruent responses. Specifically, the dual-process theory of moral behavior (Greene et al., 2001) predicts longer response time for a rational choice in response to a moral dilemma under the condition of personal involvement than for a rational choice under the condition of impersonal involvement. However, descriptives indicated that response time was longer for emotionally incongruent response only under the conditions of partial information (Fig. 2). In support, we conducted 2 × 2 × 2 × 2 ANOVA, with choice rationality (response to the task) as an additional independent variable. The results show that the main effect of accessibility, *F*(1, 283) = 8.59, *p* < .01, ε^2^ = .02, and the interaction effects of involvement by accessibility, *F*(1, 283) = 5.48, *p* < .05, ε^2^ = .01, involvement by choice rationality, *F*(1, 283) = 14.43, *p* < .001, ε^2^ = .04, and accessibility by choice rationality (rational vs. irrational choice), *F*(1, 283) = 6.72, *p* < .05, ε^2^ = .02, were significant. The main effects of choice rationality, *F*(1, 283) = 3.57, *p* > .05, ε^2^ = .01, and involvement and dilemma type were not significant, both *F* < 1, ε^2^ = .00. The following were also not significant: the two-way interaction effects: dilemma type by involvement, dilemma type by accessibility, and dilemma type by choice rationality, all *F* < 1, ε^2^ = .00; the three-way interaction effects: dilemma type by involvement by choice rationality, *F*(1, 283) = 1.07, *p* > .05, ε^2^ = .00, involvement by accessibility by choice rationality, *F*(1, 283) = 1.59, *p* > .05, ε^2^ = .00, and dilemma type by involvement by accessibility and dilemma type by accessibility by choice rationality, both *F* < 1, ε^2^ = .00; and the four-way interaction, *F* < 1, ε^2^ = .00.

**Table 3:** Descriptives for response time by involvement, accessibility, and dilemma type

Involvement	Accessibility (information)	Tr	Trolley		Footbridge	
			*M*	*SD*	*M*	*SD*
Impersonal	Partial	T	2.28	0.74	2.30	0.76
		U	13.43	13.88	13.15	9.57
	Full	T	1.85	0.52	1.89	0.54
		U	7.25	3.92	7.62	4.14
Personal	Partial	T	2.16	0.60	2.29	0.63
		U	10.51	7.25	12.19	8.76
	Full	T	1.85	0.50	1.86	0.50
		U	7.15	3.53	7.25	3.48

The frequency distribution of study time was positively skewed and this was considerably improved by logarithmic transformation

*Tr* transformation, *T* logarithmically transformed, *U* untransformed (original)

Follow-up simple-effect tests showed that for moral dilemmas with partial information, the interaction between involvement and choice rationality was significant, *F*(1, 159) = 15.60, *p* < .001, ε^2^ = .09. Unsurprisingly, further simple effects within partial information revealed that the effect of choice rationality was significant, *F*(1, 82) = 8.69, *p* < .01, ε^2^ = .09, when involvement was personal, with rational choices taking more time to make (*M*_*Ln*_ = 2.81; *SD*_*Ln*_ = .38) than irrational (*M*_*Ln*_ = 2.16; *SD*_*Ln*_ = .61); however, when involvement was impersonal, the effect was significant, *F*(1, 76) = 8.56, *p* < .01, ε^2^ = .09, with rational choices taking less time (*M*_*Ln*_ = 2.03; *SD*_*Ln*_ = .52) than irrational (*M*_*Ln*_ = 2.51; *SD*_*Ln*_ = .84).

However, simple effects showed that for moral dilemmas with full information only the effect of choice rationality was significant, *F*(1, 138) = 10.69, *p* < .01, ε^2^ = .06, with rational choices taking less time (*M*_*Ln*_ = 1.79; *SD*_*Ln*_ = .49) than irrational (*M*_*Ln*_ = 2.19; *SD*_*Ln*_ = .46). These findings suggest that any emotional interference, with rational choices taking more time to make, appears as an artifact of presenting partial information and disappears when full information is presented, with rational choices taking less time.

## Discussion

Our results reveal that variation in utilitarian accessibility produces variation in moral choices. In particular, displaying full information regarding moral actions and consequences resulted in an increase of rational choices. Moreover, the effect of utilitarian accessibility was general in that it occurred across types of involvement (both personal and impersonal) and types of dilemma (both trolley and footbridge). Previous research (e.g., Greene et al., 2001) found that people took more time to judge an action as rational when a moral dilemma was personal. However, type of dilemma and involvement were confounded (McGuire et al., 2009), and utilitarian accessibility was not manipulated.

We further examined Greene and colleagues’ (2001) claim that “emotional interference” produces longer response time for emotionally incongruent responses. This prediction was only confirmed when participants made a rational choice in response to a moral dilemma under the condition of personal involvement with partial information (e.g., judging it appropriate to push the man off the footbridge in the footbridge dilemma). In contrast, with full information presented, rational choices were made faster. Therefore, our results suggest that any emotional interference, with rational choices taking more time to make, is an artifact of presenting partial information and does not happen when full information is presented, with rational choices taking less time. Given our results, a more plausible interpretation of increased response time with rational answers under conditions of partial information is reduced utilitarian accessibility rather than “emotional interference”. When decision-makers are presented with full contextual information about a particular moral action and its consequences, the framing effect will be eliminated and mental simulation will not entertain other possible outcomes of the scenario (e.g., FeldmanHall et al., 2012). Therefore, decision-makers are more vividly confronted with the effect of the action (whether personal or impersonal). It is plausible that limited utilitarian accessibility of moral actions and consequences results in a psychological uncertainty and corresponding mental simulations (compensating for reduced accessibility of moral actions and consequences). In contrast, comprehensive information about moral actions and consequences may eliminate uncertainty, and boost utility maximization in moral choices, with rational choices taking less time. Such an interpretation might be accommodated by “situation models” (e.g., Glenberg, Meyer, & Lindem, 1987), in which linguistic descriptions are understood by simulating perceptual and motor aspects of those descriptions. Therefore, more complete descriptions may facilitate simulations by reducing uncertainty. Moreover, it is well established by behavioral science theorists that decision uncertainty induces human irrationality in choice (e.g., Kusev, van Schaik, Ayton, Dent, & Chater, 2009; Kusev, van Schaik, & Aldrovandi, 2012; Tversky & Kahneman, 1992).

Our main finding is the effect of utilitarian accessibility on judgment of appropriateness and response time. Therefore, we agree with McGuire et al.’s (2009) recommendation that “More research needs to be done at a behavioral level in order to fine-tune the questions being asked before work identifying the neural correlates of moral decision-making can be useful” (p. 580).

## Electronic supplementary material

Below is the link to the electronic supplementary material.

ESM 1 (PDF 159 kb)

## Footnotes

1 Irrational choice was the reference category and rational choice was the response category.
