# Supplementary material for: Judging the morality of utilitarian actions: How poor utilitarian accessibility makes judges irrational
Source: Psychon Bull Rev. 2016 Apr 27;23(6):1961–7. doi: 10.3758/s13423-016-1029-2 (PMC5133284; doi:10.3758/s13423-016-1029-2)
Supplement: Supplementary file 1 — (PDF 159 kb) [file 13423_2016_1029_MOESM1_ESM.pdf]

Accessibility in Moral Judgments

Supplemental materials

In the experimental conditions participants were offered the following scenarios and choice options about the appropriateness of action:

A. Moral dilemmas under partial descriptions.

Footbridge, personal, partial description:
A runaway trolley is heading down the track toward five workmen who will be killed if the trolley proceeds on its present course. You are on a footbridge over the track in between the approaching trolley and the five workmen. Next to you on this footbridge is a stranger who happens to be very large. The only way to save the lives of the five workmen is to push this stranger off the bridge and onto the track below where his large body will stop the trolley. The stranger will die if you do this, but the five workmen will be saved.

Question:
Is it appropriate for you to push the stranger on to the track in order to save the five workmen?
Yes
No

Footbridge, impersonal, partial description:
A runaway trolley is heading down the track toward five workmen who will be killed if the trolley remains on its present course. You are on a footbridge over the track between the approaching trolley and the five workmen. Next to you on this footbridge is a lone workman who is hanging on a rope, painting the bridge. The only way to save the lives of the five workmen is to hit a switch, causing the rope to lower the lone workman onto the track below, where his large body will stop the trolley. The lone workman will die if you do this, but the five workmen will be saved.

Question:
Is it appropriate for you to hit the switch in order to avoid the deaths of the five workmen?
Yes
No

Trolley, impersonal, partial description:
A runaway trolley is heading down the tracks toward five workmen who will be killed if the trolley remains on its present course. On the track extending to the left are five railway workmen. On the track extending to the right is a lone railway workman. The only way to save the lives of the five workmen is to hit a switch near the tracks that will cause the trolley to proceed to the right, where the lone workman's large body will stop the trolley. The lone workman will die if you do this, but the five workmen will be saved.
Question:
Is it appropriate for you to hit the switch in order to avoid the deaths of the five workmen?
Yes
No
Trolley, personal, partial description:

For Review Only

A runaway trolley is heading down the tracks toward five workmen who will be killed if the trolley remains on its present course. A lone workman just happens to be standing near the track and you are there. The only way to save the lives of the five workmen is to push the lone workman so that he falls onto the track, where his large body will stop the trolley. The lone workman will die if you do this, but the five workmen will be saved.

Question:
Is it appropriate for you to push the stranger on to the track in order to save the five workmen?
Yes
No

B. Moral dilemmas under full descriptions.

Footbridge, personal, full description:
A runaway trolley is heading down the track toward five workmen who will be killed if the trolley proceeds on its present course. You are on a footbridge over the track in between the approaching trolley and the five workmen. Next to you on this footbridge is a lone workman who happens to be very large. The only way to save the lives of the five workmen is to push the lone workman off the bridge and onto the track below where his large body will stop the trolley. The lone workman will die if you do this, but the five workmen will be saved. The only way to save the life of the lone workman is not to push this workman off the bridge. The five workmen will die if you do this, but the lone workman will be saved.

Question:
Choose the option which is more appropriate for you:
Sacrifice one workman in order to save five workmen
Sacrifice five workmen in order to save one workman

Footbridge, impersonal, full description:
A runaway trolley is heading down the track toward five workmen who will be killed if the trolley remains on its present course. You are on a footbridge over the track between the approaching trolley and the five workmen. Next to you on this footbridge is a lone workman who is hanging on a rope, painting the bridge. The only way to save the lives of the five workmen is to hit a switch, causing the rope to lower the lone workman onto the track below, where his large body will stop the trolley. The lone workman will die if you do this, but the five workmen will be saved. The only way to save the life of the lone workman is not to hit the switch. The five workmen will die if you do this, but the lone workman will be saved.

Question:
Choose the option which is more appropriate for you:
Sacrifice one workman in order to save five workmen
Sacrifice five workmen in order to save one workman

Trolley, impersonal, full description:
A runaway trolley is heading down the tracks toward five workmen who will be killed if the trolley remains on its present course. On the track extending to the left are five

For Review Only

railway workmen. On the track extending to the right is a lone railway workman. The only way to save the lives of the five workmen is to hit a switch near the tracks that will cause the trolley to proceed to the right, where the lone workman's large body will stop the trolley. The lone workman will die if you do this, but the five workmen will be saved. The only way to save the life of the lone workman is not to hit the switch near the tracks. The five workmen will die if you do this, but the lone workman will be saved.

Question:
Choose the option which is more appropriate for you:
Sacrifice one workman in order to save five workmen
Sacrifice five workmen in order to save one workman

Trolley, personal, full description:
A runaway trolley is heading down the track toward five workmen who will be killed if the trolley remains on its present course. A lone workman just happens to be standing near the track and you are there. The only way to save the lives of the five workmen is to push the lone workman so that he falls onto the track, where his large body will stop the trolley. The lone workman will die if you do this, but the five workmen will be saved. The only way to save the life of the lone workman is not to push this workman. The five workmen will die if you do this, but the lone workman will be saved.

Question:
Choose the option which is more appropriate for you:
Sacrifice one workman in order to save five workmen
Sacrifice five workmen in order to save one workman

For Review Only

Accessibility in Moral Judgments

Supplemental materials

In the experimental conditions participants were offered the following scenarios and choice options about the appropriateness of action:

A. Moral dilemmas under partial descriptions.

Footbridge, personal, partial description:
A runaway trolley is heading down the track toward five workmen who will be killed if the trolley proceeds on its present course. You are on a footbridge over the track in between the approaching trolley and the five workmen. Next to you on this footbridge is a stranger who happens to be very large. The only way to save the lives of the five workmen is to push this stranger off the bridge and onto the track below where his large body will stop the trolley. The stranger will die if you do this, but the five workmen will be saved.

Question:
Is it appropriate for you to push the stranger on to the track in order to save the five workmen?
Yes
No

Footbridge, impersonal, partial description:
A runaway trolley is heading down the track toward five workmen who will be killed if the trolley remains on its present course. You are on a footbridge over the track between the approaching trolley and the five workmen. Next to you on this footbridge is a lone workman who is hanging on a rope, painting the bridge. The only way to save the lives of the five workmen is to hit a switch, causing the rope to lower the lone workman onto the track below, where his large body will stop the trolley. The lone workman will die if you do this, but the five workmen will be saved.

Question:
Is it appropriate for you to hit the switch in order to avoid the deaths of the five workmen?
Yes
No

Trolley, impersonal, partial description:
A runaway trolley is heading down the tracks toward five workmen who will be killed if the trolley remains on its present course. On the track extending to the left are five railway workmen. On the track extending to the right is a lone railway workman. The only way to save the lives of the five workmen is to hit a switch near the tracks that will cause the trolley to proceed to the right, where the lone workman's large body will stop the trolley. The lone workman will die if you do this, but the five workmen will be saved.
Question:
Is it appropriate for you to hit the switch in order to avoid the deaths of the five workmen?
Yes
No
Trolley, personal, partial description:

For Review Only

A runaway trolley is heading down the tracks toward five workmen who will be killed if the trolley remains on its present course. A lone workman just happens to be standing near the track and you are there. The only way to save the lives of the five workmen is to push the lone workman so that he falls onto the track, where his large body will stop the trolley. The lone workman will die if you do this, but the five workmen will be saved.

Question:
Is it appropriate for you to push the stranger on to the track in order to save the five workmen?
Yes
No

B. Moral dilemmas under full descriptions.

Footbridge, personal, full description:
A runaway trolley is heading down the track toward five workmen who will be killed if the trolley proceeds on its present course. You are on a footbridge over the track in between the approaching trolley and the five workmen. Next to you on this footbridge is a lone workman who happens to be very large. The only way to save the lives of the five workmen is to push the lone workman off the bridge and onto the track below where his large body will stop the trolley. The lone workman will die if you do this, but the five workmen will be saved. The only way to save the life of the lone workman is not to push this workman off the bridge. The five workmen will die if you do this, but the lone workman will be saved.

Question:
Choose the option which is more appropriate for you:
Sacrifice one workman in order to save five workmen
Sacrifice five workmen in order to save one workman

Footbridge, impersonal, full description:
A runaway trolley is heading down the track toward five workmen who will be killed if the trolley remains on its present course. You are on a footbridge over the track between the approaching trolley and the five workmen. Next to you on this footbridge is a lone workman who is hanging on a rope, painting the bridge. The only way to save the lives of the five workmen is to hit a switch, causing the rope to lower the lone workman onto the track below, where his large body will stop the trolley. The lone workman will die if you do this, but the five workmen will be saved. The only way to save the life of the lone workman is not to hit the switch. The five workmen will die if you do this, but the lone workman will be saved.

Question:
Choose the option which is more appropriate for you:
Sacrifice one workman in order to save five workmen
Sacrifice five workmen in order to save one workman

Trolley, impersonal, full description:
A runaway trolley is heading down the tracks toward five workmen who will be killed if the trolley remains on its present course. On the track extending to the left are five

For Review Only

railway workmen. On the track extending to the right is a lone railway workman. The only way to save the lives of the five workmen is to hit a switch near the tracks that will cause the trolley to proceed to the right, where the lone workman's large body will stop the trolley. The lone workman will die if you do this, but the five workmen will be saved. The only way to save the life of the lone workman is not to hit the switch near the tracks. The five workmen will die if you do this, but the lone workman will be saved.

Question:
Choose the option which is more appropriate for you:
Sacrifice one workman in order to save five workmen
Sacrifice five workmen in order to save one workman

Trolley, personal, full description:
A runaway trolley is heading down the track toward five workmen who will be killed if the trolley remains on its present course. A lone workman just happens to be standing near the track and you are there. The only way to save the lives of the five workmen is to push the lone workman so that he falls onto the track, where his large body will stop the trolley. The lone workman will die if you do this, but the five workmen will be saved. The only way to save the life of the lone workman is not to push this workman. The five workmen will die if you do this, but the lone workman will be saved.

Question:
Choose the option which is more appropriate for you:
Sacrifice one workman in order to save five workmen
Sacrifice five workmen in order to save one workman

For Review Only
